# The clock modulator Nobiletin mitigates astrogliosis-associated neuroinflammation and disease hallmarks in an Alzheimer’s disease model

**DOI:** 10.1096/fj.202101633R

**Published:** 2022-03

**Authors:** Marvin Wirianto, Chih-Yen Wang, Eunju Kim, Nobuya Koike, Ruben Gomez-Gutierrez, Kazunari Nohara, Gabriel Escobedo, Jong Min Choi, Chorong Han, Kazuhiro Yagita, Sung Yun Jung, Claudio Soto, Hyun Kyoung Lee, Rodrigo Morales, Seung-Hee Yoo, Zheng Chen

**Affiliations:** 1 Department of Biochemistry and Molecular Biology, The University of Texas Health Science Center at Houston (UTHealth), Houston, Texas, USA; 2 Department of Pediatrics, Baylor College of Medicine, Neurological Research Institute, Texas Children’s Hospital, Houston, Texas, USA; 3 Department of Physiology and Systems Bioscience, Graduate School of Medicine, Kyoto Prefectural University of Medicine, Kyoto, Japan; 4 Department of Neurology, The University of Texas Health Science Center (UTHealth), Houston, Texas, USA; 5 Department of Cell Biology, Genetics and Physiology, Faculty of Sciences, University of Malaga, Malaga, Spain; 6 Department of Biochemistry and Molecular Biology, Baylor College of Medicine, Houston, Texas, USA; 7 Centro Integrativo de Biologia Y Quimica Aplicada (CIBQA), Universidad Bernardo O’Higgins, Santiago, Chile

**Keywords:** Alzheimer’s disease, Aβ pathology, circadian clock, neuroinflammation, Nobiletin (NOB), ROR nuclear receptors

## Abstract

Alzheimer’s disease (AD) is a devastating neurodegenerative disorder, and there is a pressing need to identify disease-modifying factors and devise interventional strategies. The circadian clock, our intrinsic biological timer, orchestrates various cellular and physiological processes including gene expression, sleep, and neuroinflammation; conversely, circadian dysfunctions are closely associated with and/or contribute to AD hallmarks. We previously reported that the natural compound Nobiletin (NOB) is a clock-enhancing modulator that promotes physiological health and healthy aging. In the current study, we treated the double transgenic AD model mice, APP/PS1, with NOB-containing diets. NOB significantly alleviated β-amyloid burden in both the hippocampus and the cortex, and exhibited a trend to improve cognitive function in these mice. While several systemic parameters for circadian wheel-running activity, sleep, and metabolism were unchanged, NOB treatment showed a marked effect on the expression of clock and clock-controlled AD gene expression in the cortex. In accordance, cortical proteomic profiling demonstrated circadian time-dependent restoration of the protein landscape in APP/PS1 mice treated with NOB. More importantly, we found a potent efficacy of NOB to inhibit proinflammatory cytokine gene expression and inflammasome formation in the cortex, and immunostaining further revealed a specific effect to diminish astrogliosis, but not microgliosis, by NOB in APP/PS1 mice. Together, these results underscore beneficial effects of a clock modulator to mitigate pathological and cognitive hallmarks of AD, and suggest a possible mechanism via suppressing astrogliosis-associated neuroinflammation.

## 1 | INTRODUCTION

Alzheimer’s disease (AD) is a devastating age-associated neurodegenerative disease characterized by gradual decline in memory and cognitive functions.^1,2^ The classical pathological hallmarks of the disease include the extracellular deposition of amyloid-β (Aβ) plaques and the intracellular accumulation of hyperphosphorylated tau proteins forming neurofibrillary tangles.^3,4^ Aβ peptides (most commonly 40 and 42 amino acids in length) are produced by sequential cleavage of β-secretase (BACE1) and γ-secretase of the amyloid-β precursor protein (APP). Aβ deposition promotes tau pathological development, together driving neurodegeneration.^2^ Many cellular processes have been proposed to act upstream as pathogenic mechanisms. In particular, mounting evidence indicates a key role of neuroinflammation during AD disease progression, as astrogliosis and microgliosis, namely, reactive activation of the glia cells astrocytes and microglia, are commonly found in AD brains.^5–7^ Astrocytes play multiple important roles in neuronal health, including metabolic support, glymphatic flow, and neuroinflammatory response.^8^ Astrogliosis occurs in response to brain injury including neurodegeneration, and, under adverse conditions, can lead to neurotoxic consequences such as impaired Aβ clearance and elevated neuroinflammation.^8,9^ Microglia are the key player in the brain innate immune system, functioning to engulf Aβ peptides to restrict pathology and to direct systemic neuroinflammatory responses through cytokine release.^2,10^

Accumulating evidence supports a close relationship between AD and disruption of sleep/wake cycles and circadian rhythms.^3,11,12^ AD patients are known to suffer marked sleep fragmentation and nocturnal activity/day-time sleepiness; in advanced stages, the severe disruption or reversal of normal sleep cycles constitutes the primary cause for institutionalization.^12^ Prospective studies showed that degeneration of circadian activity patterns and/or sleep fragmentation occur in the early, presymptomatic phase during AD pathogenesis, and displayed predictive values for later development of cognitive deficits, pathological Aβ deposition, and dementia.^13,14^ In mice and humans, levels of Aβ and tau in interstitial fluid or cerebrospinal fluid, respectively, were found to fluctuate during the sleep/wake cycle, peaking in the active phase, and sleep deprivation and sleep-promoting orexin signaling were found to exert opposing effects on these molecular markers.^15–19^ Multiple lines of evidence also directly link circadian clocks with AD.^10,11,20^ An intact circadian oscillator, containing positive (CLOCK, BMAL1, RORs) and negative (PERs, CRYs, REV-ERBs) core clock components,^21^ is required for neuronal maintenance and function, cognitive functions, and behavior.^22^ Importantly, circadian disruption by genetic mutations or environmental factors leads to neurodegeneration and impaired cognitive functions.^11^ For example, genetic ablation of BMAL1, an essential transcription factor in the positive arm of the core loop, led to pronounced astrogliosis and exacerbated amyloid burden.^22,23^ Using viral or conditional knockout strategies, circadian disruption in different brain cell types, including neuron, astrocytes, and microglia, have been shown to adversely aggravate pathological, neuroinflammatory, or cognitive hallmarks in AD mouse models.^24–26^ Finally, in studies of AD models and human samples, various abnormalities in circadian rhythms and oscillatory gene expression have been discovered, including alterations in expression level, phase, or amplitude.^27–29^ Together, these observations suggest that the circadian/sleep cycles, including the core oscillator itself, may be modifiable factors involved in AD pathogenesis.

Given the regulatory functions of the circadian clock in various cellular and physiological processes known to be dysregulated in AD,^10–12^ manipulating the clock or clock components may modify AD symptoms and progression. The clock governs tissue-specific gene expression^30,31^; in particular, a number of AD-related genes display circadian oscillatory expression under normal conditions, and core oscillator components were found to directly bind to promoter elements of some of these genes.^17,23,30^ In addition to sleep as a clock regulated physiological function,^32,33^ growing evidence strongly indicates a role of the clock in the immune system, governing innate and adaptive immunity in both peripheral tissues and the brain.^26,34,35^ Accordingly, a number of interventional strategies targeting these clock-associated processes have been applied to AD and other neurodegenerative diseases, including bright light, melatonin, and more recently time-restricted feeding.^2,10,12^ Several clock-targeting drugs have also been tested in animal models. In addition to sleep-promoting orexin agonists,^15,16^ studies have examined effects of compounds directly acting on core oscillator components. For example, suppression of REV-ERBs, key components in the secondary loop of the core oscillator, by a chemical inhibitor has been found to promote microglial phagocytosis of fibrillary Aβ1–42, enhancing its clearance in 5xFAD mice.^36^ In accordance, an agonist of REV-ERBs showed the effects of increased neuroinflammation and exaggerated cognitive deficits in APP knock-in mice.^37^ Together, these studies suggest a promising strategy to target the circadian machinery, including core oscillator components, in order to improve AD-related pathology and other disease hallmarks.

We previously identified a natural compound called Nobiletin (NOB) as an agonist of the RORs nuclear receptor, key clock components functioning in the secondary loop of the oscillator to antagonize REV-ERBs.^38^ We showed that NOB was able to activate circadian clocks to improve various metabolic and physiological functions in disease and aged mice.^38–42^ Interestingly, NOB has been shown to exert a broad spectrum of beneficial effects in rodents, including various AD models.^43,44^ For example, NOB was recently found to improve memory functions in LPS-treated WT C57B/6J mice, associated with reduced microglial production of proinflammatory cytokines.^45^ In light of our discovery of circadian targeting by NOB and the emerging link between the clock and amyloid pathology and neuroinflammation, we investigated the effects and mechanisms of NOB using the double transgenic AD model mice, APP/PS1. In a recent study focusing on female APP/PS1 mice,^46^ we found significant effects of NOB to modulate cortex clock gene expression and Aβ pathology. In the current work, we characterized AD hallmarks in male APP/PS1 mice, and further investigated circadian and neuroinflammatory functions of NOB. Our results identified astrogliosis as an important pathophysiological target of NOB, suggesting a propitious clock-based intervention to ameliorate AD hallmarks.

## 2 | MATERIALS AND METHODS

### 2.1 | Animals studies

The APP/PS1 double transgenic mice (JAX 034829) were bred with B6C3F1/J (JAX 100010) to generate APP/PS1 and WT littermates as previously described.^46^ All mice were maintained under the 12:12 h light:dark (LD) cycles unless otherwise indicated. Zeitgeber time (ZT) 0 and 12 represent light on (7 am) and off (7 pm), respectively. At 3–4 months of age, mice were treated with regular diets containing macronutrients at equivalent levels with Purina 5053, with or without 0.1% Nobiletin (Research Diets, Inc., NJ, USA). NOB was obtained from commercial sources (GenDEPOT and Selleck Chemicals, both in Houston, Texas, USA). The treatment continued until mice were sacrificed at 18–20 months of age. Mice were used for multiple experiments, starting with the least invasive and with ample recovery time (more than 3 weeks) between experiments. All animal husbandry and experimentation were approved by UTHealth Center for Laboratory Animal Medicine and Care (CLAMC) and were conducted in compliance with IACUC guidelines.

### 2.2 | Immunohistochemistry

Immunohistochemical analyses were performed as previously reported.^47,48^ Mouse brain tissues were placed in 10% neutral buffered formalin overnight, followed by paraffin embedding and sectioning. Sagittal sections of the brain were stained for Aβ with the 4G8 antibody (#800701, Biolegend, CA, USA). Sections were visualized by using DAB substrate kit with nickel (Vector Laboratories, CA, USA) and mounted using DPX mounting medium (Electron Microscopy Sciences). Approximately 4–6 slices per animal were taken and analyzed in the ImageJ (NIH) software and quantified using burden threshold.

### 2.3 | Real-time PCR analysis

RT-qPCR analysis was carried out as previously described.^46^ Total RNA was isolated from frozen cortex powder using PureXtract RNAsol (GenDEPOT, TX, USA) and 1 μg of RNAs were used to synthesize cDNA. Gene expressions were analyzed by using Mx3000p (Agilent technologies, CA, USA). All gene expressions were normalized relative to *Gapdh*. Primer sequences are shown in Table S1.

### 2.4 | Immunoblotting

Immunoblotting were performed as previously described.^49^ Commercial antibodies were used to detect NLRP3 (Cell Signaling, MA, USA), GAPDH (Abcam, Cambridge, UK), and RhoA (Abclonal, MA, USA). Quantification of immunoblot gels from 3 independent experiments was done by using ImageJ (NIH).

### 2.5 | Immunofluorescence staining

Immunofluorescence staining was performed by using paraffinized slices as described previously.^47^ Sagittal sections of mouse brain were stained with anti-GFAP (Z033401–2, Agilent Technologies, CA, USA) and anti-S100β (GA50461–2, Agilent Technologies) antibodies for astrocytes and an anti-IBA1 antibody (Fujifilm, Tokyo, Japan) for microglia. Stained slices were mounted with DAPI-Fluoromount-G (SouthernBiotech). Fluorescence was acquired by using the Axiocam 506 mono (Carl Zeiss, Oberkochen, Germany) equipped with an inverted 63× 0.45NA UPLFL objective on an Axio Imager M2 Upright Microscope (Carl Zeiss). Images were taken in the channel sequence of FITC (Ex 493, Em 528), DAPI (Ex 390, Em 435), and Texas Red (Ex 576, Em 603). Six to ten locations in each slide were selected for the quantification. To examine astrocyte morphology/hypertrophy, GFAP signal area for individual astrocytes was measured as cell size, and the average area for each cell process was measured to determine GFAP fiber thickness. To measure astrocyte cell number, S100β^+^ cells were counted in cortex and hippocampus images.

### 2.6 | Proteomics analysis

Proteomics was performed as previously described with modifications.^50^ Briefly, frozen mouse brain samples were lysed in 50 mM ammonium bicarbonate by repeated thawing-boiling-freezing and followed by trypsin digestion. Digested peptides were separated into 5 pools by offline high pH reverse phase chromatography as described previously.^50^ The pooled samples were analyzed on Orbitrap Fusion Tribrid system (Thermo Fisher Scientific) coupled with an Easy-nLC 1000 nanoflow LC system (Thermo Fisher Scientific). The instrument was operated in data-dependent mode, acquiring fragmentation spectra of the top 50 strongest ions. Parent mass spectrum was acquired in the Orbitrap, and higher-energy collisional dissociation (HCD) fragmented MS/MS spectrum was acquired in rapid mode. Obtained spectra were searched and validated by using Proteome Discoverer 2.1 interfaced with Mascot algorism (Mascot 2.4, Matrix Science) and grouped to gene products (GPs) by in-house gpGrouper algorithm.^51^ GP quantification was performed using the label-free, intensity-based absolute quantification (iFOT). Data were analyzed and visualized with in-house Tackle platform and iPathway guide.^52^ Functional analysis was performed using Metascape.^53^

### 2.7 | Novel object recognition test

Novel object recognition assays were performed as described previously.^54,55^ Briefly, 15-month old male mice (*n* = 7–10) were habituated for 3 consecutive days for 5 min each within an empty Plexiglas arena (45 × 25 × 20 cm) before trial. During the training phase, mice were placed in the arena alongside two identical objects placed at the opposite end and were allowed to explore for 10 min. After a 24-h delay, mice were presented with a novel object of similar dimension alongside the familiar object and were allowed to explore for 5 min. The trial was measured and recorded using TopScan Suite software (CleverSys Inc.). Specifically, the Plexiglas arena is divided into four identical quadrants, with two quadrants containing objects. The behavior was scored as an exploration when the mouse entered the quadrants to explore objects for 20 s or longer. The arena was cleaned between trials to remove residual scent. The discrimination ratio refers to percentage of time that mice explores the novel object.

### 2.8 | Circadian and sleep analyses

Circadian and sleep assays were conducted as previously described.^56,57^ Briefly, 16-month old male mice (*n* = 7–10) were single housed in wheel-running cages in a 12:12 h LD controlled cabinet (300 lux, room temperature at 22.6–24.1°C and relative humidity 38%–42%). After 3 weeks of acclimation, animals were subjected to constant darkness (DD). Free-running period analysis was performed during 21 days in DD. Wheel-running data were extracted and analyzed by using Clocklab Analysis software (Actimetrics).

For sleep analysis, 10-month old male mice (*n* = 7–10) were tested in a noninvasive piezoelectric transducer sleep/wake recording system (Signal Solutions) as previously described.^39,57^ Before testing, mice were acclimated for 2 days in individually housed cages with free access to food and water under 12:12 h LD cycle. The initial 48 h acclimation period was followed by actual data recording for 5 days. Data were extracted and analyzed by using Sleepstats software (Signal Solutions).

### 2.9 | Metabolic chamber analysis

Briefly, 12–13 months old mice (*n* = 7–10) were individually housed in metabolic chambers with free access to food and water as previously described.^46^ Measurements of oxygen consumption, carbon dioxide production, and heat production were recorded every 8–12 min for three days.

### 2.10 | Statistical analysis

Results are presented as mean ± SEM unless otherwise stated. Data were analyzed using student’s *t*-test or ANOVA (including one-way, 2-way and 3-way as indicated) followed by post-hoc analysis using Tukey’s multiple comparison test using GraphPad Prism. A value of *p* < .05 was considered statistically significant.

## 3 | RESULTS

### 3.1 | Mitigation of AD hallmarks by NOB in male APP/PS1 mice

**FIGURE 1:**
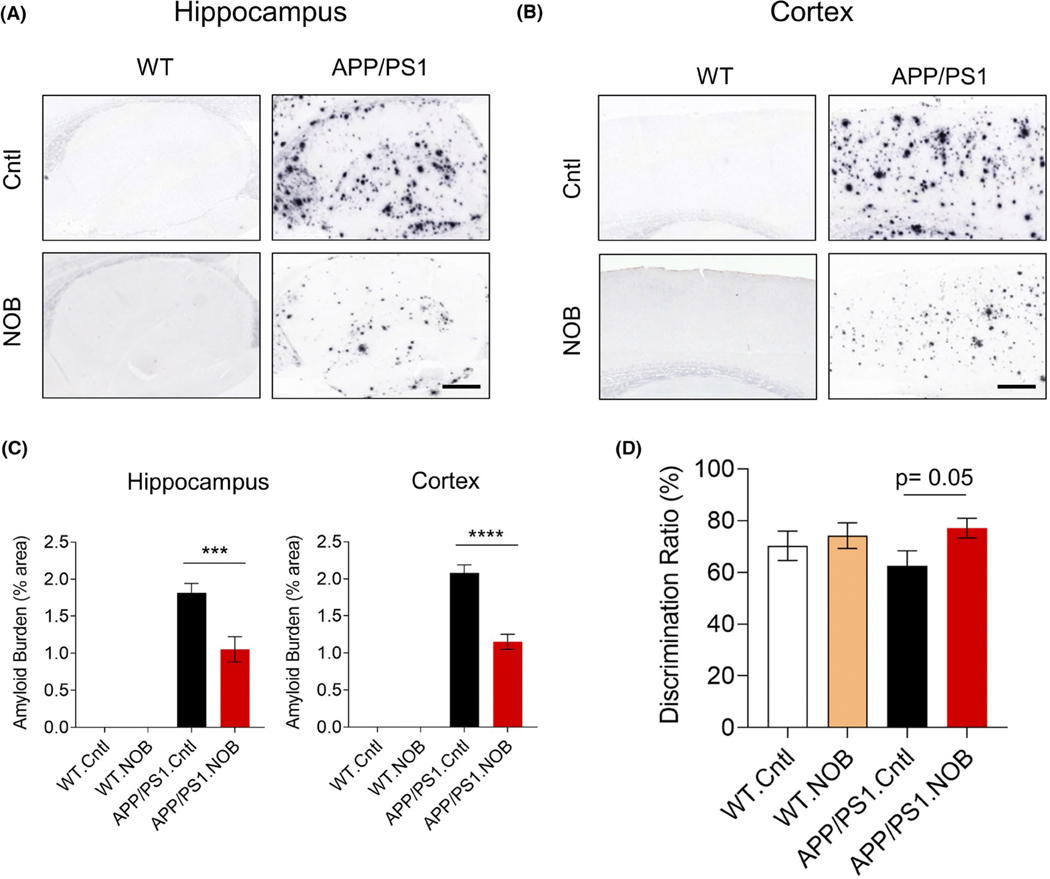
NOB mitigates Aβ pathology and recognition memory in APP/PS1 mice. (A,B) Immunohistochemistry of Aβ deposition using the 4G8 antibody in (A) the hippocampus and (B) the cortex. (C) Quantification of Aβ burden. Error bars represent mean ± SEM. Two-way ANOVA shows significant statistical difference between APP/PS1.Cntl and APP/PS1.NOB in the hippocampus (***p < .001) and cortex (****p < .0001). Scale bar, 500 μm. (D) Discrimination ratio of novel object recognition test. T-test shows a trend between APP/PS1. Cntl and APP/PS1.NOB (*p* = .05)

We first evaluated effects of NOB on Aβ pathology and cognitive function in male APP/PS1 mice using a chronic treatment regimen. Male WT and APP/PS1 mice at 3–4 months of age were fed with regular diets with or without NOB, and several circadian and physiological parameters were monitored (see below) before sacrifice at 18–20 months of age. At the end point, immunohistochemistry analysis was performed using both hippocampus and cortex sections. Whereas APP/PS1 mice showed significant Aβ plaque accumulation in both brain regions (Figure 1A,B), NOB treatment was able to markedly ameliorate this phenotype, reducing plaque burden by 42.2% and 44.7%, respectively (Figure 1C). In accordance with this efficacy of NOB on Aβ pathology, we also observed an improvement in AD-associated cognitive behavior. Specifically, treated mice at 15 months of age were subjected to the well-established novel object recognition (NOR) test.^54^ NOB showed a trend to improve the recognition memory in APP/PS1 mice as evidenced by the normalized discrimination ratio relative to WT mice (Figure 1D). These results together indicate mitigating effects of NOB to improve pathological and behavioral hallmarks of AD.

### 3.2 | NOB regulates circadian gene expressions in APP/PS1 mice

Our previous studies revealed a clock-modulatory role of NOB as an agonist of the ROR receptors in the core oscillator, thereby regulating target gene expression and down-stream metabolic and physiological processes.^38,39^ During the treatment period, we conducted several assays to investigate the effects of NOB on circadian behavior and systemic metabolism. As shown in Figure S1A, we did not observe significant changes in the circadian free-running period among the mouse groups, regardless of NOB treatment. In the piezo sleep monitoring, APP/PS1.Cntl mice showed significantly lower sleep qualities based on total sleep duration, and number of daytime bouts compared to WT.Cntl, which was not improved by NOB treatment in APP/PS1 mice (Figure S1B). Likewise, metabolic cage measurements (Figure S1C) showed that NOB improved respiratory parameters in WT but not APP/PS1 mice.

**FIGURE 2:**
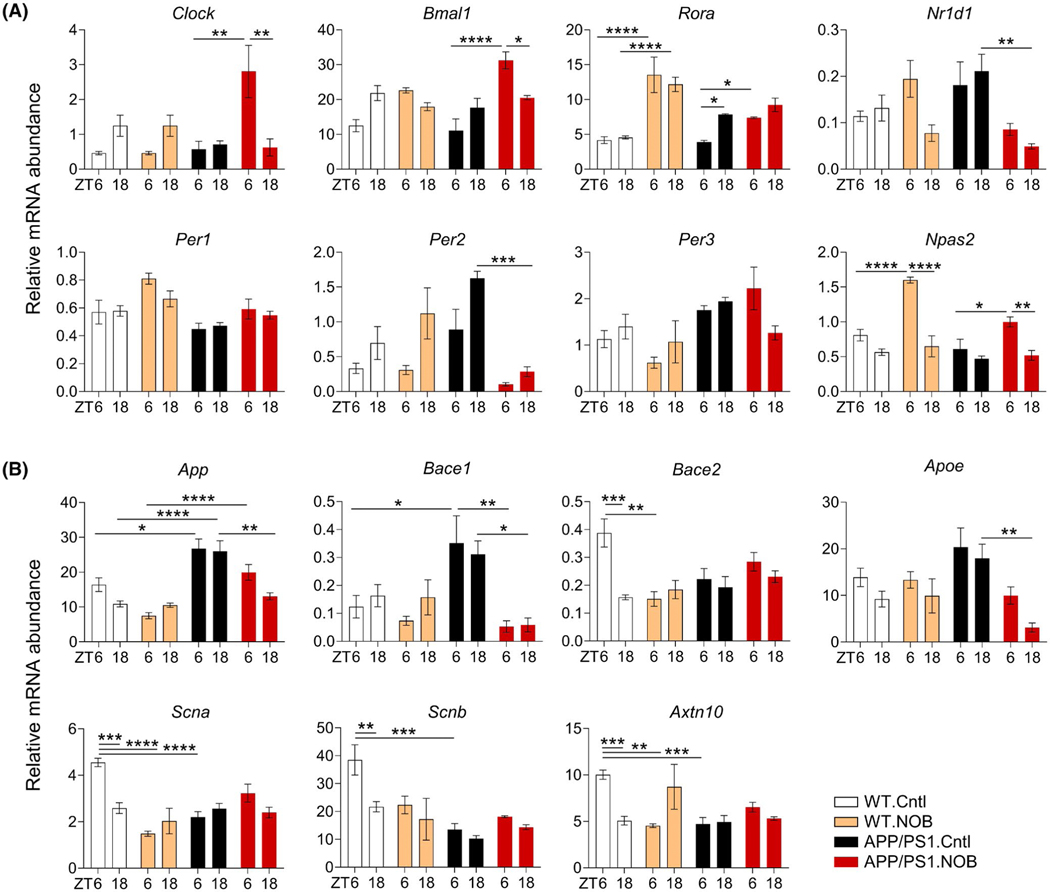
NOB modulates mRNA expressions of core clock genes and AD-related genes in WT and APP/PS1 mice. mRNA expressions of (A) core clock genes and (B) AD-related genes in cortex tissues were measured by using real-time qPCR (*n* ≥ 3/each group). Data are presented as mean ± SEM in bar graph. *p < .05, **p < .01, ***p < .001, ****p < .0001, three-way ANOVA with Tukey’s multiple comparisons. Statistical significance and F distribution of interaction are shown in Table S2

We next investigated specific effects of NOB on cortex expression of core clock genes and clock-controlled genes in cortex tissues collected at zeitgeber time (ZT) 6 and 18. Several clock genes showed significantly different expressions between WT and APP/PS1, including *Rora*, *Per1*, *Per3*, and *Npas2* (Figure 2 and Table S2). Expressions of a number of core clock genes were found to be altered by NOB (Figure 2A, Figure S2A) in a genotype- and circadian time-dependent manner. For example, NOB was found to reduce *Per2* expression in APP/PS1 mice, with no effects in WT. Furthermore, the temporal variation in the expression of ROR target genes such as *Clock*, *Bmal1*, and *Npas2* was significantly increased by NOB in APP/PS1 mice compared to APP/PS1.Cntl. In comparison, the overall expression levels of *Per2* and *Nr1d1*, both functioning in the negative arm, were markedly reduced by NOB in APP/PS1 mice. These results suggest NOB modulates the diurnal expression of ROR target genes, and differentially affects genes in the positive and negative arms of the oscillator.

Previous studies suggest a regulatory role of the clock in AD gene expression.^17,23,30^ As expected, expression of *App* is higher in APP/PS1 mice relative to WT (*p* < .05 at ZT6 and *p* < .0001 at ZT18), due to transgene expression, which was partly reduced by NOB, especially at ZT18 (*p* < .01) (Figure 2B). In addition, *Bace1* and *Apoe* expression, also elevated in APP/PS1 compared to WT without treatment, was decreased by NOB in APP/PS1 mice (*p* < .05 for *Bace1* at ZT18, *p* < .01 for *Bace1* at ZT6 and *Apoe* at ZT18). Furthermore, APP/PS1 mice showed a trend of altered expression of several other AD-related genes such as *Scna*, *Scnb*, and *Axtn10* by NOB treatment in APP/PS1 mice (Figure 2B). These results are consistent with a role of NOB to regulate time-dependent expression of AD-related genes, including those required for Aβ production, in the cortex of APP/PS1 mice.

### 3.3 | NOB significantly alters protein expression in APP/PS1 mice

**FIGURE 3:**
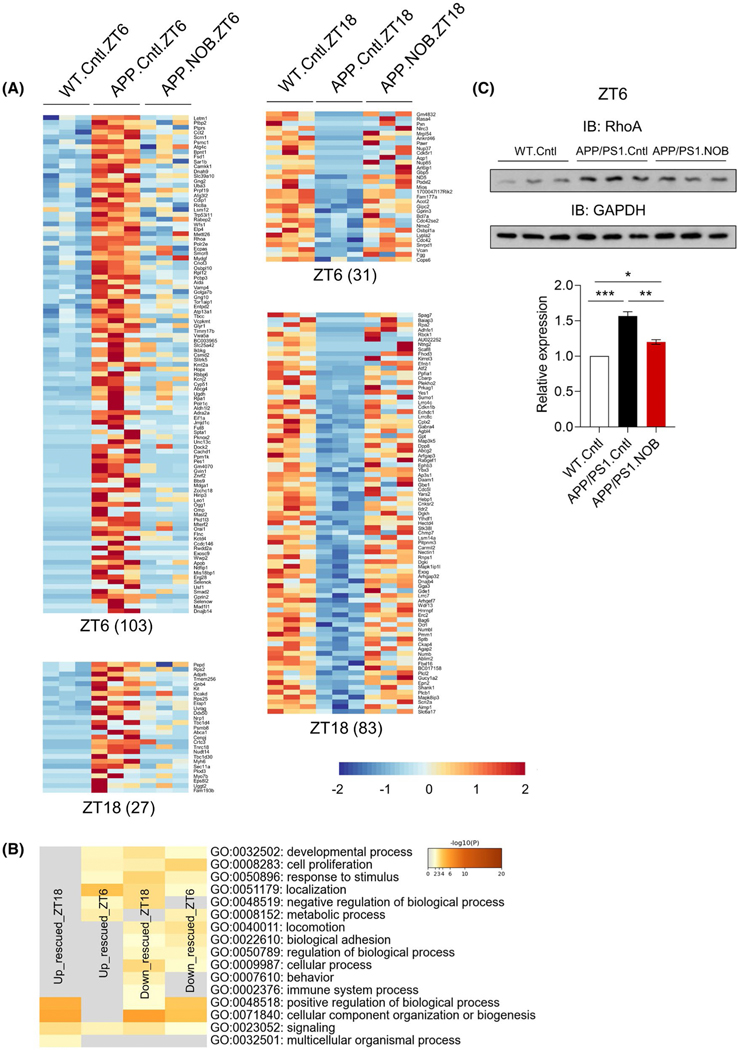
NOB alters cortical proteomic landscape in APP/PS1 mice. (A) Heat map view of the upregulated (left) and downregulated (right) proteins in APP/PS1 mice in ZT6 and ZT18. Each protein is represented as a horizontal line, ordered vertically by log2 fold change in expression level of WT.Cntl relative to APP/PS1 in both ZT6 and ZT18. (B) Heat map showing the top enrichment clusters by Metascape analysis of upregulated and downregulated proteins in APP/PS1 cortex and rescued by NOB treatment at two circadian time points (ZT6 and ZT18). (C) Immunoblotting of RhoA protein in WT.Cntl, APP/PS1.Cntl and APP/PS1.NOB in the cortex at ZT6. Bottom panel: Quantification of the RhoA expression in the cortex at ZT6. Data are presented as mean ± SEM in bar graph. One-way ANOVA shows significant statistical difference between WT.Cntl, APP/PS1.Cntl and APP/PS1.NOB (*p < .05, **p < .01; ***p < .001)

We performed cortical proteomic analysis to broadly survey the alteration in protein landscape, again using samples collected at ZT6 and ZT18. We first conducted analysis at each time point. We found 103 and 27 differentially expressed proteins (DEPs) with elevated levels, and 31 and 83 DEPs with diminished amounts in APP/PS1 compared with WT, which was normalized by NOB to varying degrees at ZT6 and ZT18, respectively (Figure 3A). Metascape analysis revealed significant enrichment in cellular pathways including cell proliferation, metabolism, and immune responses (Figure 3B). Among these proteins whose abundance is altered by AD and NOB, 37 have been described in literature to be AD-related (Figure S3A). As a validation, we performed immunoblotting on one of the AD-related proteins, the small GTPase RhoA previously shown to play varying roles in tau phosphorylation and neurodegeneration.^58,59^ The levels of RhoA in the cortex was found to be upregulated in APP/PS1 and downregulated by NOB (Figure 3C).

Next, to delineate circadian time-dependent effects, we analyzed diurnal patterns of differentially expressed proteins. As shown in the Venn diagram (Figure S3B), circadian DEPs in WT.Cntl (199), APP/PS1.Cntl (335), and APP/PS1.NOB (224) show very small overlaps (WT.Cntl/APP/PS1.Cntl:14, WT.Cntl/APP/PS1.NOB: 7, APP/PS1. Cntl/APP/PS1.NOB: 9). Metascape analysis of these circadian DEPs revealed several enriched pathways, including protein membrane localization and autophagy pathways in WT.Cntl, receptor tyrosine kinase signaling in APP/PS1.Cntl and behavior and mRNA metabolic process in APP/PS1.NOB (Figure S3C). Overall these results indicate NOB restored protein landscape in APP/PS1 mice in a circadian time-dependent manner.

### 3.4 | NOB reduces levels of proinflammatory cytokines and NLRP3 inflammasomes in APP/PS1 mice

**FIGURE 4:**
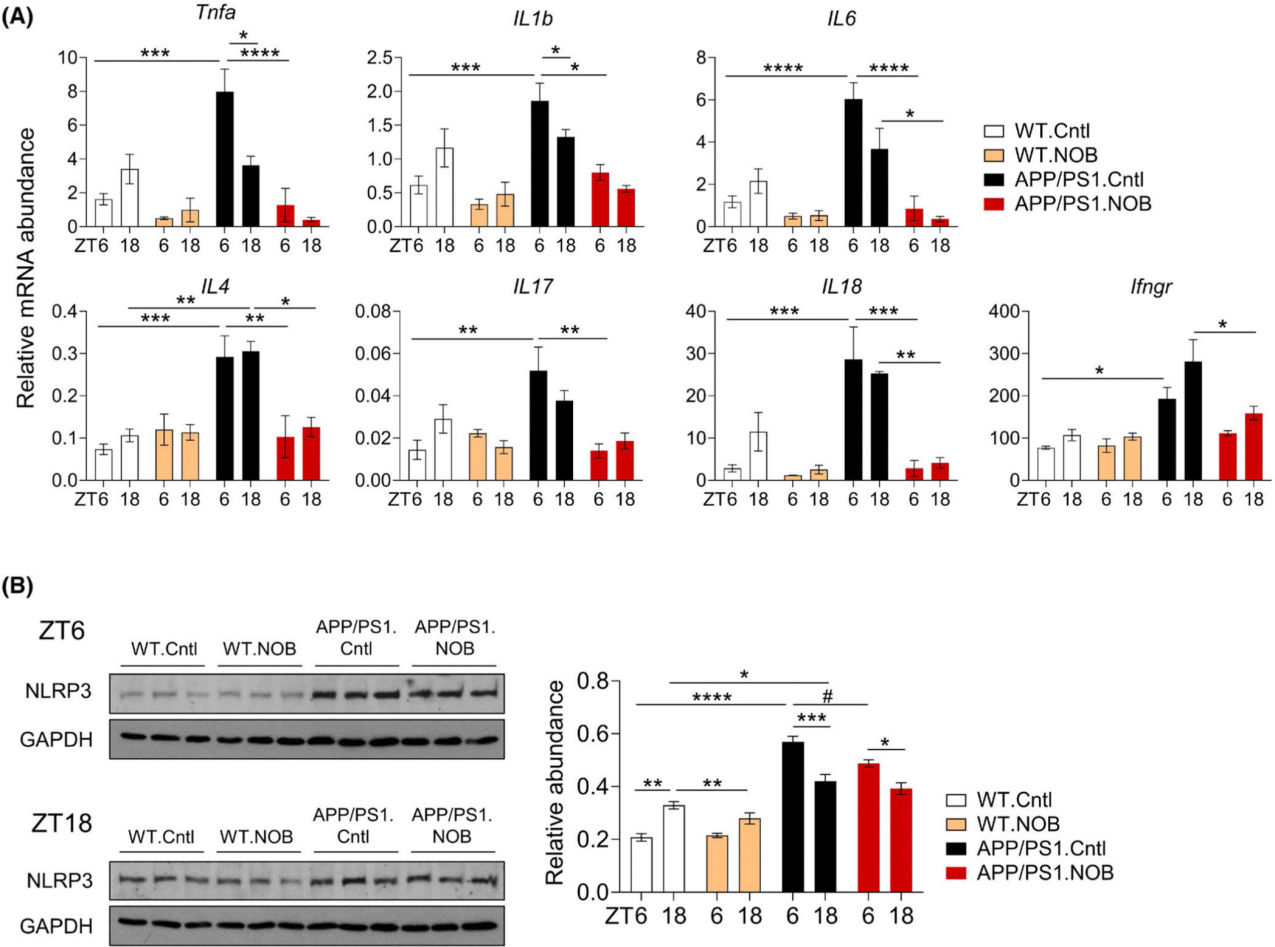
NOB reduces proinflammatory cytokine gene expression and NLRP3 protein levels in the cortex of APP/PS1 mice. (A) RT-qPCR analysis of mRNA expressions of proinflammatory cytokines in cortex tissues collected at ZT6 and ZT18 (*n* ≥ 3/each group). Data are presented as mean ± SEM in bar graph. *p < .05, **p < .01, ***p < .001, ****p < .0001, three-way ANOVA with Tukey’s multiple comparisons. (B) NLRP3 expression in cortex tissues were measured by western blot (*n* = 3/each group). Left panel shows blot images and right panel shows the quantification at ZT6 and ZT18. Data are presented as mean ± SEM in bar graph. *p < .05, **p < .01, ****p < .0001, three-way ANOVA with Tukey’s multiple comparisons. (^#^*p* < .05), one-way ANOVA with Tukey multiple comparisons. Statistical significance and *F* distribution of interaction are shown in Table S3

Neuroinflammation is a key mechanism contributing to AD pathology.^6,7^ Previously, it has been shown that APP/PS1 mice express higher levels of proinflammatory cytokines and inflammasome.^60–62^ We therefore investigated NOB effects on cytokine gene expression and inflammasome formation using cortex samples (Figure 4). As expected, expressions of proinflammatory cytokine genes such as *Tnfa*, *Il1b*, *Il6*, *Il4*, *Il17*, *Il18*, and *Ifngr* were markedly elevated in APP/PS1.Cntl compared to WT.Cntl, particularly at ZT6. Whereas, we did not observe significant effect of NOB on cytokine gene expression in WT mice, the exaggerated cytokine gene expressions were markedly reduced by NOB treatment in APP/PS1 mice (Figure 4A and Table S3). We next investigated the protein level of NLRP3, an essential component of the inflammasome.^63^ NLRP3 exhibited circadian time-dependent expressions in the WT (compare ZT6 and ZT18, Figure 4B); consistent with the cytokine results above, NLRP3 protein level was upregulated in APP/PS1.Cntl compared to WT.Cntl, but NOB decreased inflammasome expression significantly at ZT6 (*p* < .05) (Figure 4B). These results illustrate an antineuroinflammatory function of NOB, suppressing proinflammatory cytokines and inflammasomes in the cortex tissue.

### 3.5 | NOB strongly ameliorates reactive astrogliosis

**FIGURE 5:**
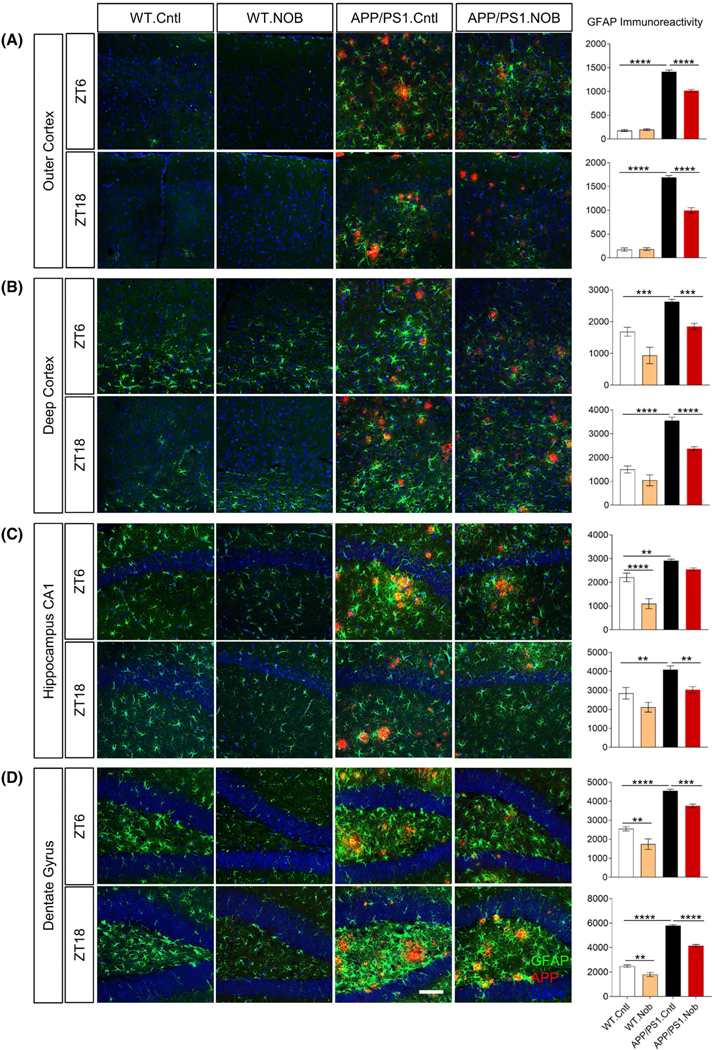
NOB significantly reduces reactive astrocytes in APP/PS1 hippocampus. Double immunofluorescence of astrocytes (GFAP, green) and Aβ (4G8, red) in APP/PS1 mice with DAPI (blue) in the (A) outer cortex, (B) deep cortex, (C) hippocampus CA1, (D) dentate gyrus at two different time points (ZT6 and ZT18). Scale bar: 100 μm. Right Panels: Quantification of the GFAP immunoreactivity in the different areas of the cortex and hippocampus. Two-way ANOVA shows significant statistical difference between APP/PS1.Cntl and APP/PS1.NOB (**p < .01; ***p < .001; ****p < .0001). This analysis revealed significant effects for interaction (treatment × genotype) as follows. Figure 5A: ZT6, *F*(1,29) = 45.63, *p* < .0001; ZT18, *F*(1,29) = 50.39, *p* < .0001. Figure 5B: ZT18, *F*(1,29) = 5.924, *p* < .05. Figure 5C: ZT6, *F*(1,29) = 9.133, *p* < .01. Figure 5D: ZT18, *F*(1,30) = 15.76, *p* < .001

To further delineate the cellular basis underlying the NOB mitigation of neuroinflammation, we examined astrocytes and microglia, the two main glia cell types involved in neuroinflammation as well as Aβ clearance through phagocytosis and degradation.^6–8,64^ During AD progression, neuroinflammation and neurodegeneration are associated with reactive astrogliosis, often characterized by cellular hypertrophy and increased Glial fibrillar acidic protein (GFAP) expression.^65–67^ To examine NOB effects on astrogliosis, we performed 4G8 and GFAP double immunofluorescence staining using brain sections. As shown in Figure 5, GFAP immunoreactivity was significantly increased in the outer and deep cortex, CA1 in the hippocampus, and the dentate gyrus of APP/PS1. Cntl mice relative to the WT. NOB treatment led to significant reductions in GFAP signals in all APP/PS1 brain areas examined at both ZT6 and ZT18, with the exception of CA1 at ZT6 (Figure 5C). NOB was also able to reduce GFAP activation in the CA1 (ZT6) and the dentate gyrus (both ZT6 and ZT18) in the WT mice (Figure 5C,D). As a control, we quantified Aβ pathology by 4G8 immunofluorescence staining, and found significant reductions in both total and sized based plaques compared to APP/PS1. Cntl mice (Figure S4). These results are consistent with our immunohistochemistry results in Figure 1. To further evaluate effects of NOB on astrogliosis, we analyzed astrocyte cell morphology (a key hallmark) and counted S100β-positive astrocytes at ZT6. Whereas GFAP-positive astrocytes in untreated APP/PS1 mice showed significantly increased process thickness as well as cell size relative to those in untreated WT mice, indicating astrogliosis, NOB treatment markedly reduced astrocyte hypertrophy in both cortex and hippocampus regions (Figure S5A,B). Furthermore, NOB showed a trend to reduce the number of S100β (another astrocyte marker) positive astrocytes in APP/PS1 brain (Figure S5C,D). These results together indicate a strong effect of NOB to ameliorate astrogliosis.

We next investigated whether NOB affects microgliosis. Previous studies have reported that production of pro-inflammatory cytokines such as TNFα, IL1β, and IL6 by hippocampal microglia shows rhythmic oscillation throughout the day,^64^ and targeting the circadian component REV-ERBs, antagonistic to RORs, leads to improved Aβ removal by microglia.^36^ We performed IBA1 and 4G8 double immunofluorescence staining to visualize microglia adjacent to Aβ plaques (Figure S6). Whereas, APP/PS1 mice showed significantly increased expression of Iba1 nearby the plaques as expected, NOB did not lead to significant reduction in microgliosis, suggesting a predominant effect of NOB on astrocytes, not microglia, in APP/PS1 mice.

## 4 | DISCUSSION

Given the massive social and medical burden of AD and the lack of effective regimens for prevention and therapy, there is a pressing need to understand disease-modifying factors and explore novel therapeutic strategies. Circadian timing is closely linked to AD; for example, sundowning, manifested as agitation or delirium in the evening, is a common symptom in AD patients, especially in the mid-disease stage.^68,69^ More importantly, presymptomatic circadian dysfunctions predict severity of AD hallmarks in humans,^13,14^ and mouse studies demonstrate disease causality of circadian disruption in AD models.^70^ We previously identified a clock-enhancing small molecule NOB and reported its beneficial effects to promote health and healthy aging.^38,39,71^ In the current study using male APP/PS1 mice, we show that NOB markedly improved Aβ pathology in both the hippocampus and the cortex, importantly accompanied by a trend of enhanced cognitive memory in APP/PS1 mice. Note that the cognitive test was performed at 15 months of age, whereas previous studies have employed an earlier time window (12−13 months of age) for APP/PS1 mice.^72,73^ Future studies will determine whether tests performed during an earlier time window or in other AD models may reveal more pronounced effects of NOB in cognition. While we did not observe significant alteration in circadian wheel-running activity, sleep, or systemic metabolism, gene expression analysis and proteomic profiling in the cortex reveal significant and broad alterations in expression of clock and clock-controlled genes in a circadian time-dependent manner. These observations are consistent with our recent study in female APP/PS1 mice,^46^ where we also observed more pronounced circadian time-dependent effects in the cortex than systemic parameters, suggesting a predominantly local effect of NOB targeting tissue-specific regulatory networks. Importantly, we discovered a strong effect of NOB to mitigate neuroinflammation, including reduced proinflammatory cytokine gene expression and inflammasome formation as evidenced by decreased NLRP3 levels. Somewhat surprisingly, immunostaining studies revealed a specific role of NOB to suppress astrogliosis, but not microgliosis. Together, our study highlights a clock-modulatory compound as a promising anti-AD agent, via a mechanism of mitigating astrogliosis-associated neuroinflammation.

NOB is a natural polymethoxylated compound with excellent pharmacokinetic profiles and demonstrated efficacies against various diseases and pathologies.^74–76^ NOB has also been applied to various neurological disease models, including AD. For example, in 3XTg AD mice, NOB was found to reduce soluble Aβ1–40 levels in the brain and partly rescued cognitive deficits in Y-maze and novel object tests.^77^ Despite a well-established anti-inflammatory role of NOB, only a few studies thus far have investigated its efficacy to counter neuroinflammation in cells and WT C57B/6J mice, but not in AD models.^43,45^ Our current study extends these prior observations. We report herein the functional effects of NOB to ameliorate Aβ pathology and improve recognition memory, and importantly demonstrate a specific role to mitigate astrogliosis-associated neuroinflammation. At the molecular level, we previously reported the ROR receptors in the core circadian oscillator as the direct target of NOB.^38^ As the clock plays a ubiquitous regulatory role in cellular and physiological processes, we propose that this clock-targeting activity of NOB may serve as a unifying mechanism contributing to its numerous beneficial effects.^71^ Consistently, we observed circadian time-dependent alteration in the clock/ROR-controlled gene expression and inflammatory markers in NOB-treated mice relative to the control. The beneficial effects of NOB as an agonist of RORs are consistent with previous studies showing concordant effects on AD pathology and neuroinflammation by chemical targeting of REV-ERBs, the opposing nuclear receptors of RORs.^36,37^ For example, inhibiting REV-ERBs by its antagonist SR8278 was shown to promote Aβ phagocytosis by microglia, thus improving amyloid plaque pathology.^36^ However, the functional outcomes of such chemical modulators can vary depending on the context and assays used, as another study showed that activation of REV-ERBs by its agonist SR9009 reduced LPS-induced neuroinflammation in the hippocampus.^78^

In recent years, active research has illustrated important yet complex roles of neuroinflammation in AD pathogenesis and progression.^6,7^ Microglia, the innate immune cells in the brain, are believed to play an important role in the pathophysiology of this disease.^2,79^ Phagocytosed Aβ has been shown to activate microglial inflammasomes, leading to elevated levels of proinflammatory cytokines and oxidative stress and contributing to tau pathology and neuronal loss. Astrocytes also play an important role in cytokine production and Aβ clearance through the glymphatic system.^8,80^ It has been shown that activated microglia can trigger activation of a neurotoxic subtype of astrocytes by secreted proinflammatory cytokines, leading to neurodegeneration.^9^ Somewhat surprisingly, we observed robust mitigation of astrogliosis by NOB; in contrast, microgliosis was largely unaltered. The reason for this specific effect on astrocyte activation is currently unclear. Given the heterogeneous nature and context-dependent neurotoxic or neuroprotective effects of glia cells,^79,81^ the subtypes of activated astrocytes/microglia need to be further investigated. Since the current analysis is limited to the endpoint, a longitudinal study of astrocyte and microglia activation would provide important insight into the dynamic process of neuroinflammatory response in NOB-treated APP/PS1 mice. Finally, since circadian pathways are highly cell type-specific,^30,31^ NOB may elicit distinct circadian reprogramming of gene expression in neurons, astrocytes, and microglia. Future studies are required to address these possibilities.

The circadian and molecular mechanisms underlying NOB effects against AD remain to be elucidated. We initially discovered Nobiletin (NOB) as a clock-enhancing compound and, including the current study, have investigated its in vivo efficacy in several disease and aging models known to exhibit dampened rhythms.^38–42,46,71^ Future studies utilizing a full circadian time course (6 time points or more over the circadian cycle) are needed to delineate the specific effects of NOB on transcriptomic oscillation in APP/PS1 mice, including circadian and clock-regulated AD-related genes described herein.^3,10,11^ NOB appears to exert genotype-specific effects, with more pronounced changes observed in APP/PS1 compared to WT (e.g., proinflammatory cytokine expression in Figure 4). This is consistent with our previous studies,^38,39^ where strong beneficial effects of NOB were observed in disease and aged mice whereas control mice that are young and healthy with a robust clock showed little or much diminished response to NOB. These and other observations that NOB efficacy is best manifested in a compromised condition (disease or aging) are significant because the clock is tightly regulated under normal conditions and plays a key role in physiological homeostasis.^21,82^ While it is beneficial to restore circadian robustness in pathological conditions, exaggeration of normal oscillatory amplitude may have deleterious consequences (e.g., morning spike of blood pressure). While it is interesting to speculate that NOB specifically targets disease/aging conditions by rejuvenating the clock, in-depth mechanistic studies are required to delineate the cellular reprogramming leading to improved tissue and systemic functions.

In conclusion, our study illustrates a promising efficacy of the clock-modulating compound NOB to alleviate astrogliosis and neuroinflammation, likely contributing to improved Aβ pathology and recognition memory. Future studies should further delineate circadian and cellular mechanisms underlying its potent antineuroinflammatory function against AD.

## Supplementary Material

SuppInfo published

## Abbreviations:

Aβ: amyloid-β
AD: Alzheimer’s disease
APP: amyloid-β precursor protein
APP/PS1: APP/Presenilin double mutant mouse
BACE1: β-secretase
BMAL1: brain muscle ARNT-like
CLAMC: Center for Laboratory Animal Medicine and Care
CLOCK: Circadian locomotor output cycles kaput
CRYs: cryptochrome1/2
DD: constant darkness
FAD: familial Alzheimer’s disease
GFAP: glial fibrillar acidic protein
LD: light:dark
NOB: Nobiletin
NOR: novel object recognition
PERs: period1/2/3
REV-ERBs: Nr1d1 (reverse of erb)
RORs: RAR-related orphan receptors
ZT: zeitgeber time
