# Supplementary material for: The clock modulator Nobiletin mitigates astrogliosis‐associated neuroinflammation and disease hallmarks in an Alzheimer’s disease model
Source: FASEB J. 2022 Feb 4;36(3):e22186. doi: 10.1096/fj.202101633R (PMC8887996; doi:10.1096/fj.202101633R)
Supplement: Supplementary file 1 — Supplementary Material [file FSB2-36-0-s001.pdf]

Supplemental Information

The clock modulator Nobiletin mitigates astrogliosis-associated neuroinflammation and disease hallmarks in an Alzheimer's disease model

Marvin Wirianto[1], Chih-Yen Wang[2], Eunju Kim[1], Nobuya Koike[3], Ruben Gomez-Gutierrez[4,5], Kazunari Nohara[1], Gabriel Escobedo Jr.[4], Jong Min Choi[6], Chorong Han[1], Kazuhiro Yagita[3], Sung Yun Jung[6], Claudio Soto[4], Hyun Kyoung Lee[2], Rodrigo Morales[4,7], Seung-Hee Yoo[1], Zheng Chen[1§]

1. Department of Biochemistry and Molecular Biology, The University of Texas Health Science Center at Houston (UTHealth), Houston, TX 77030.
2. Department of Pediatrics, Baylor College of Medicine, Neurological Research Institute, Texas Children's Hospital, Houston, Texas 77030
3. Department of Physiology and Systems Bioscience, Graduate School of Medicine, Kyoto Prefectural University of Medicine, Kyoto, Japan
4. Department of Neurology, The University of Texas Health Science Center (UTHealth), Houston, TX, 77030, USA
5. Department of Cell Biology, Genetics and Physiology, Faculty of Sciences, University of Malaga, Malaga, Spain
6. Department of Biochemistry and Molecular Biology, Baylor College of Medicine, Houston, TX 77030.
7. Centro Integrativo de Biologia Y Quimica Aplicada (CIBQA), Universidad Bernardo O'Higgins. Santiago, Chile.

This SI file contains 6 supplemental figures (Figure S1-S6) with figure legends and 3 supplemental table (Table S1-S3).

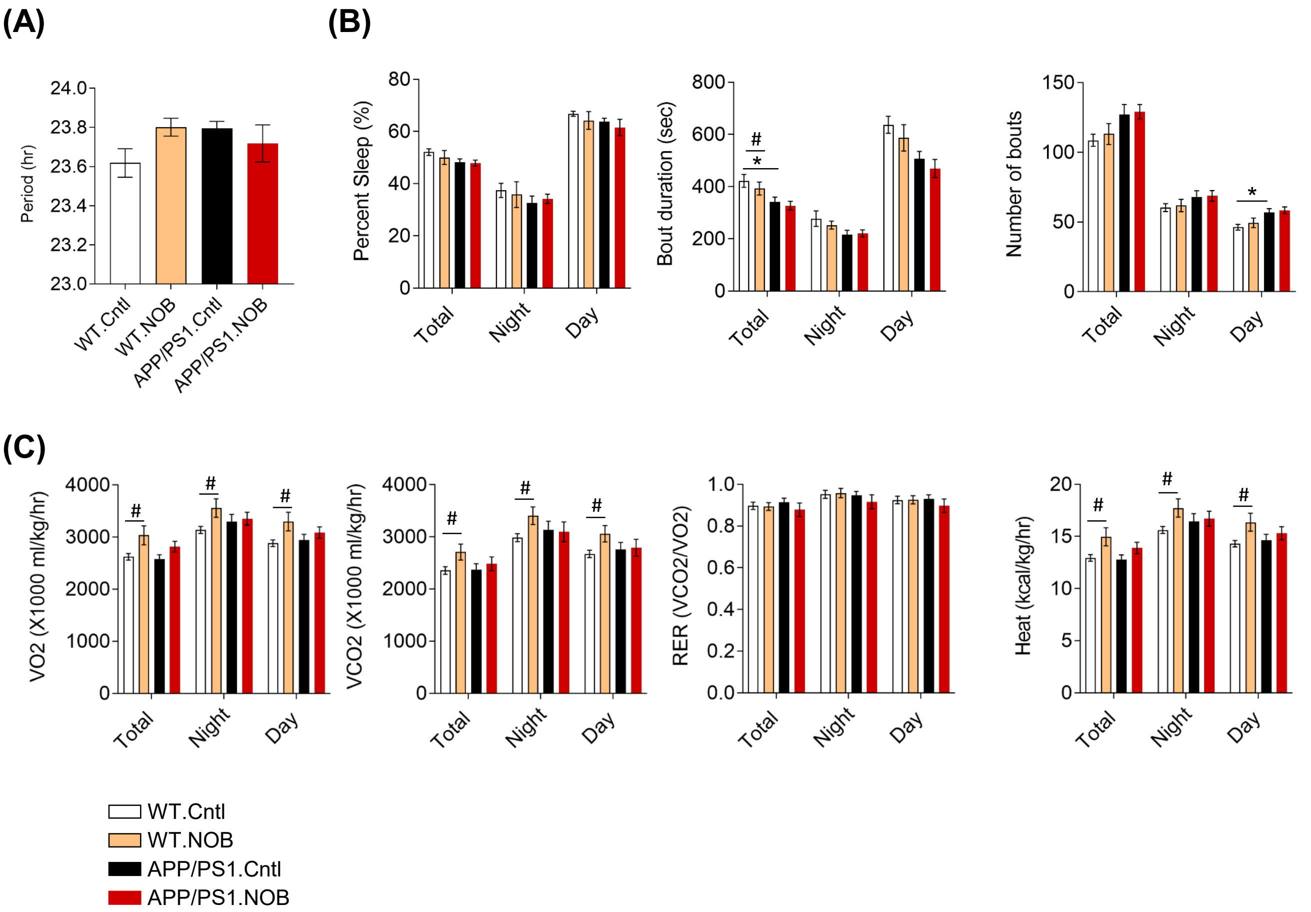

Figure S1. Characterization of circadian free-running behavior, sleep and systemic metabolism in control or NOB-treated WT and APP/PS1 mice. (A) Circadian period measurements. (B) Piezo sleep measurements. Left to right panels: percent sleep, sleep bout duration, and number of bouts. Data are presented as mean ± SEM. *, $p<0.05$: two-way ANOVA with Tukey's multiple comparisons indicating significant difference between WT.Cntl and APP/PS1.Cntl. #, $p<0.05$: t-test showing significant difference between WT.Cntl and WT.NOB. (C) Metabolic chamber measurements. Left to right: average of oxygen consumption, carbon dioxide production, respiratory exchange ratio (RER) and heat production. Data are presented as mean ± SEM. #, $p<0.05$: t-test showing significant statistical difference between WT.Cntl and WT.NOB.

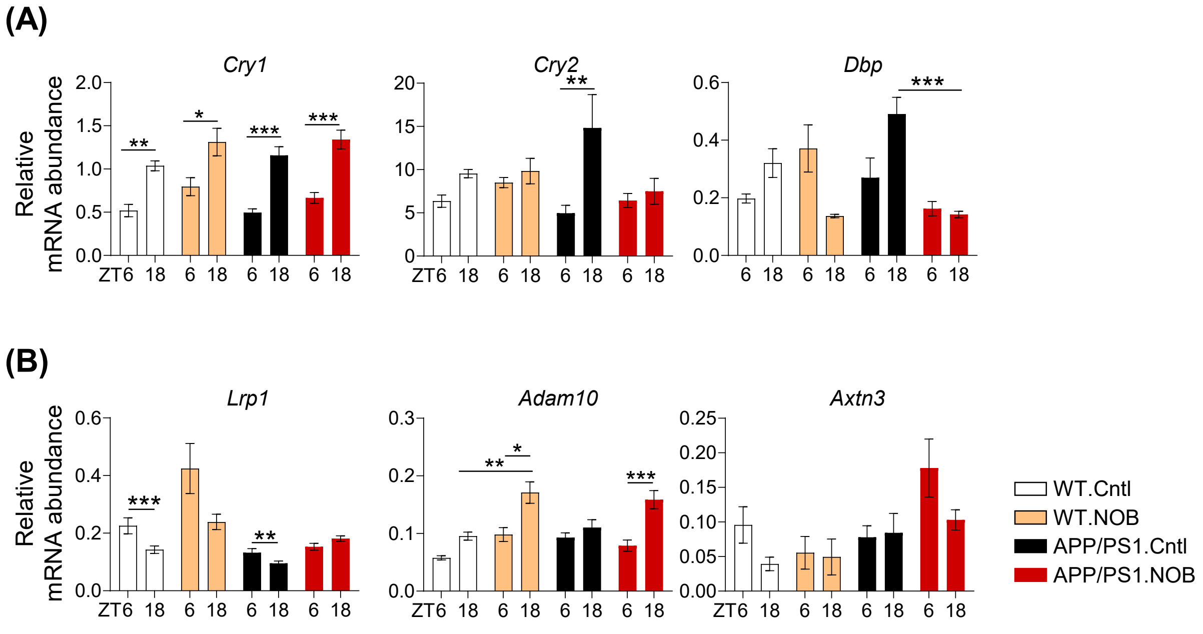

Figure S2. Cortical mRNA expressions of core clock genes and AD-related genes in WT and APP/PS1 mice. RT-qPCR analysis of (A) core clock genes and (B) AD-related genes in cortex tissues collected at ZT6 and ZT18 (n ≥ 3/each group). Data are presented as mean ± SEM in bar graph. *, p<0.05; **, p<0.01; ***, p<0.001: three-way ANOVA with Tukey's multiple comparisons. Statistical significance and F distribution of interaction are shown in Table S2.

(A)

**Proteins upregulated in APP/PS1 (103+27)**	CAMKK1, CSMD2, ENTPD2, LETM1, NDFIP1, OGG1, PCBP3, RhoA, RPL12, RWDD2a, UBA3, USF1, WFS1, ZNRF2, CENPJ, KIT
**Proteins downregulated in APP/PS1 (31+83)**	ABCG2, ABLIM2, AGAP2, AGBL4, ARFGAP3, BAG6, CKAP4, CPLX2, DAAM1, DNAJB4, ERC2, EXOG, FBXL16, GGA3, LRRC7, MAP3K5, MAPK8IP3, NECTIN1, PLCB1, RABEFF1, SUMO1

(B)

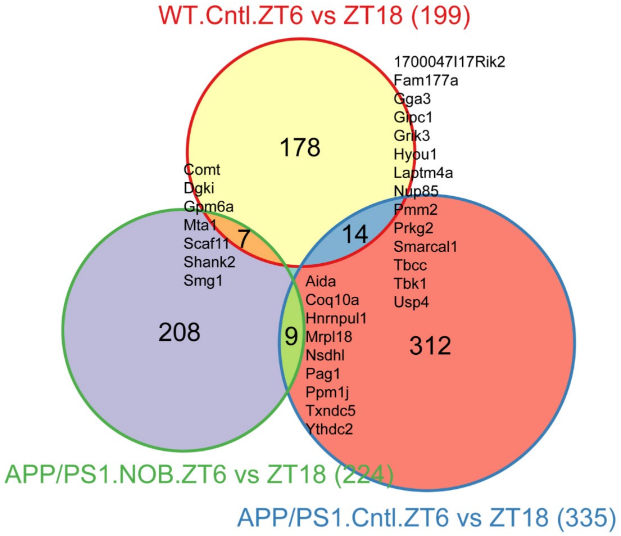

(C)

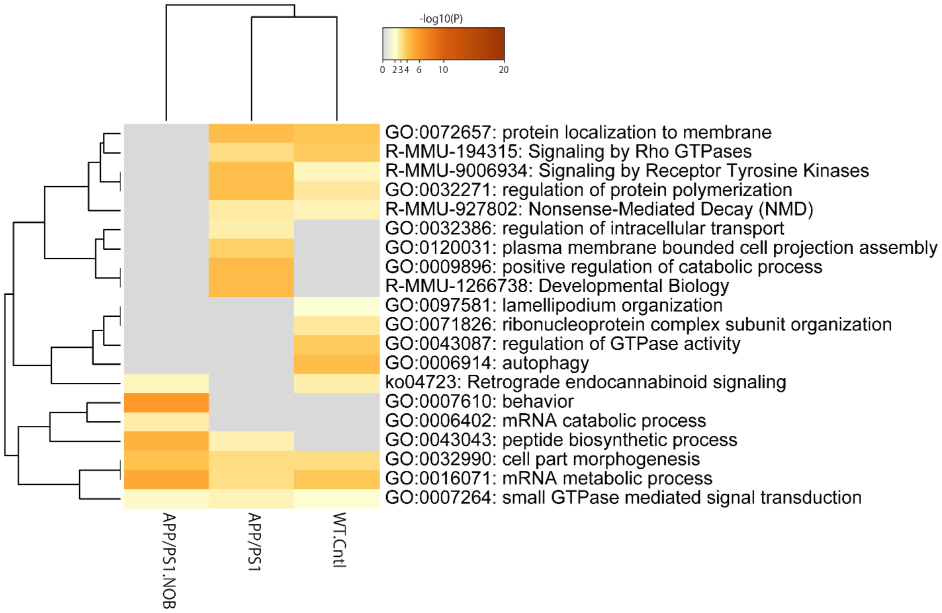

Figure S3. NOB alters proteomic landscape in the cortex. (A) List of AD-related proteins upregulated and downregulated in APP/PS1 and rescued by NOB treatment. (B) Venn Diagram of differentially expressed proteins in WT.Cntl (Top), APP/PS1.Cntl (Right) and APP/PS1 NOB (Left) groups. “WT.Cntl ZT6 vs ZT18 (199)”: Yellow circle indicates differentially expressed proteins in WT.Cntl at two circadian time points (ZT6 and ZT18). “APP/PS1 NOB ZT6 vs 18 (224)”: Purple circle indicates differentially expressed proteins in APP/PS1.NOB at two circadian time points (ZT6 and ZT18). “APP/PS1 ZT6 vs 18 (335)”: Red circle indicates differentially expressed proteins in APP/PS1.Cntl at two circadian time points (ZT6 and ZT18). (C) Heat map showing the top enrichment clusters by Metascape analysis in WT.Cntl, APP/PS1.Cntl, APP/PS1.NOB cortex at two circadian time points (ZT6 and ZT18).

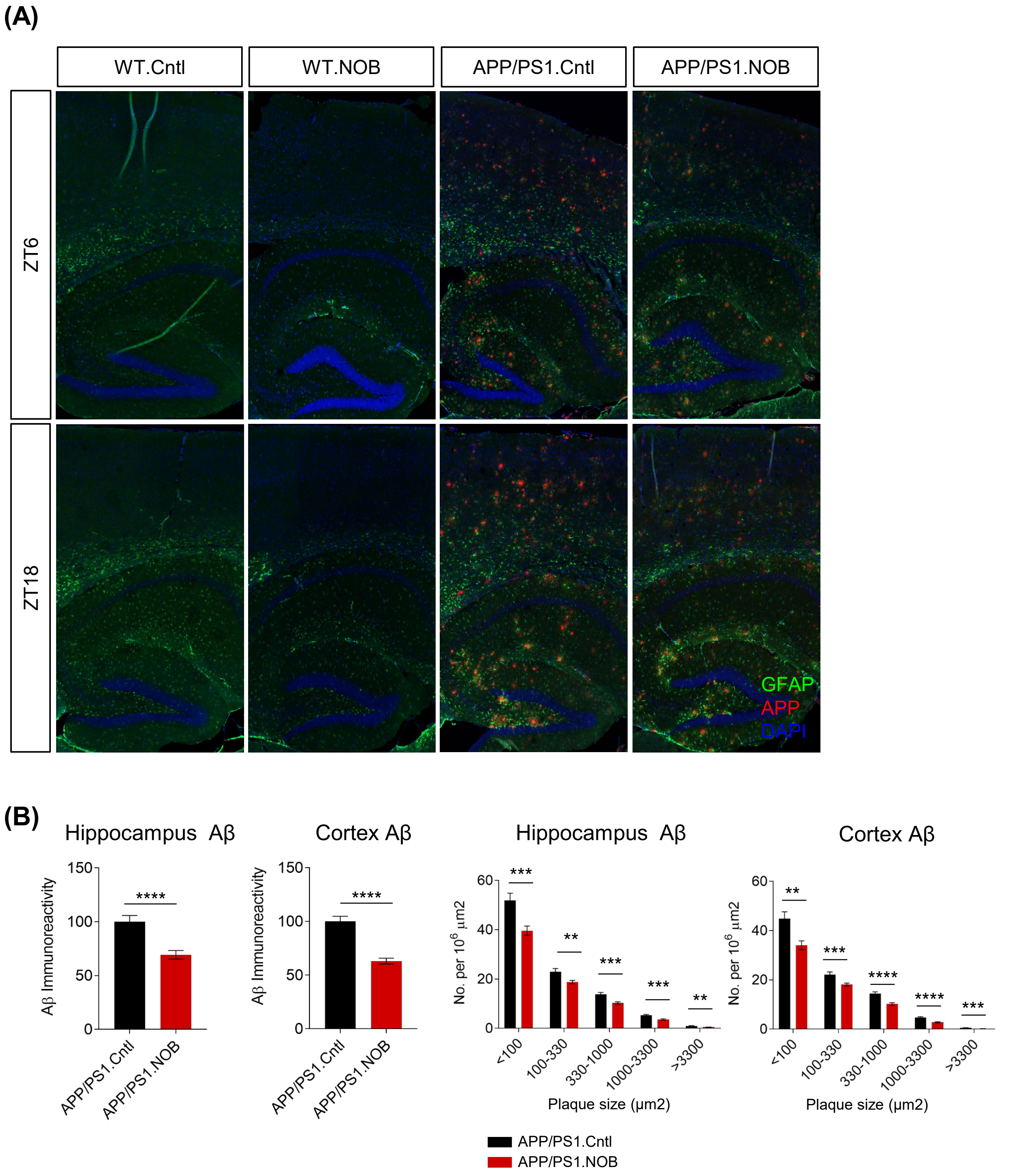

Figure S4. Immunofluorescence staining revealed diminished Aβ pathology by NOB. (A) Double immunofluorescence of brain regions containing both the cortex and the hippocampus. Scale bar: 500 μm. Green: GFAP (astrocyte); red: 4G8 (Aβ); DAPI (blue). (B) Quantification of 4G8 immunofluorescence in the cortex and the hippocampus. Right panels: Quantifications based on plaque size. T-test shows significant statistical difference between APP/PS1.Cntl and APP/PS1.NOB (**, $p<0.01$; ***, $p<0.001$; ****, $p<0.0001$).

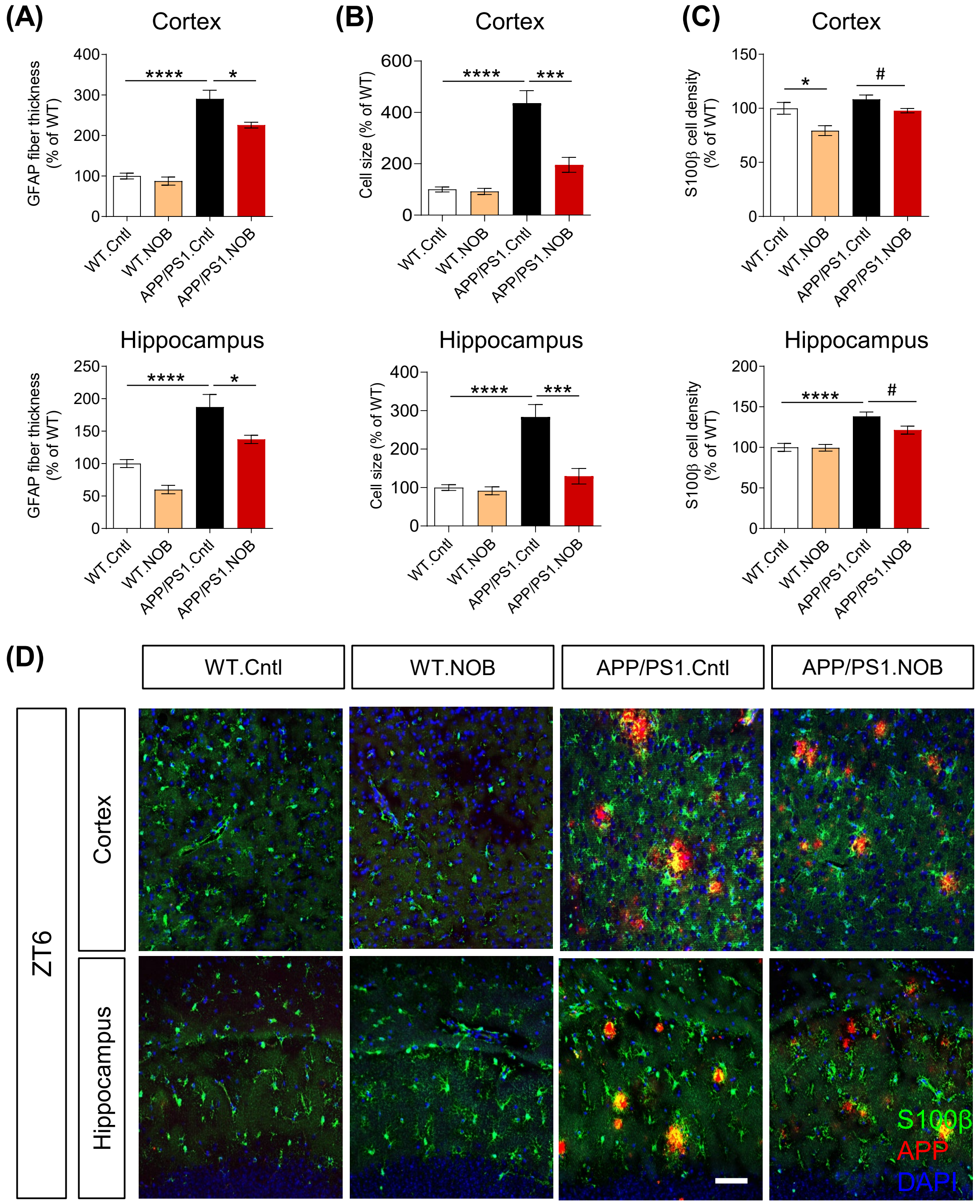

Figure S5. NOB significantly affects astrocyte cell morphology and density. (A) Process thickness of GFAP+ astrocytes in the cortex (top) and hippocampus (bottom) at ZT6. Data are presented as mean ± SEM. *, $p<0.05$; ****, $p<0.0001$: two-way ANOVA with Tukey's multiple comparisons. (B) Quantification of GFAP+ astrocyte cell size. Data are presented as mean ± SEM. ***, $p<0.001$; ****, $p<0.0001$: two-way ANOVA with Tukey's multiple comparisons. This analysis revealed a significant effect for interaction (treatment × genotype): cell size in the cortex, $F(1,21)=13.54$, $p<0.01$; cell size in the hippocampus, $F(1,21)=11.34$, $p<0.01$. (C-D) S100β immunostaining of the cortex and the hippocampus regions. (C) Quantification data are presented as mean ± SEM. *, $p<0.05$; ****, $p<0.0001$: two-way ANOVA with Tukey's multiple comparisons. #, $p<0.05$: t-test showing significant difference between APP/PS1.Cntl and APP/PS1.NOB. (D) Representative images. Scale bar: 100 µm. Green: S100β (astrocyte); red: 4G8 (Aβ); blue: DAPI.

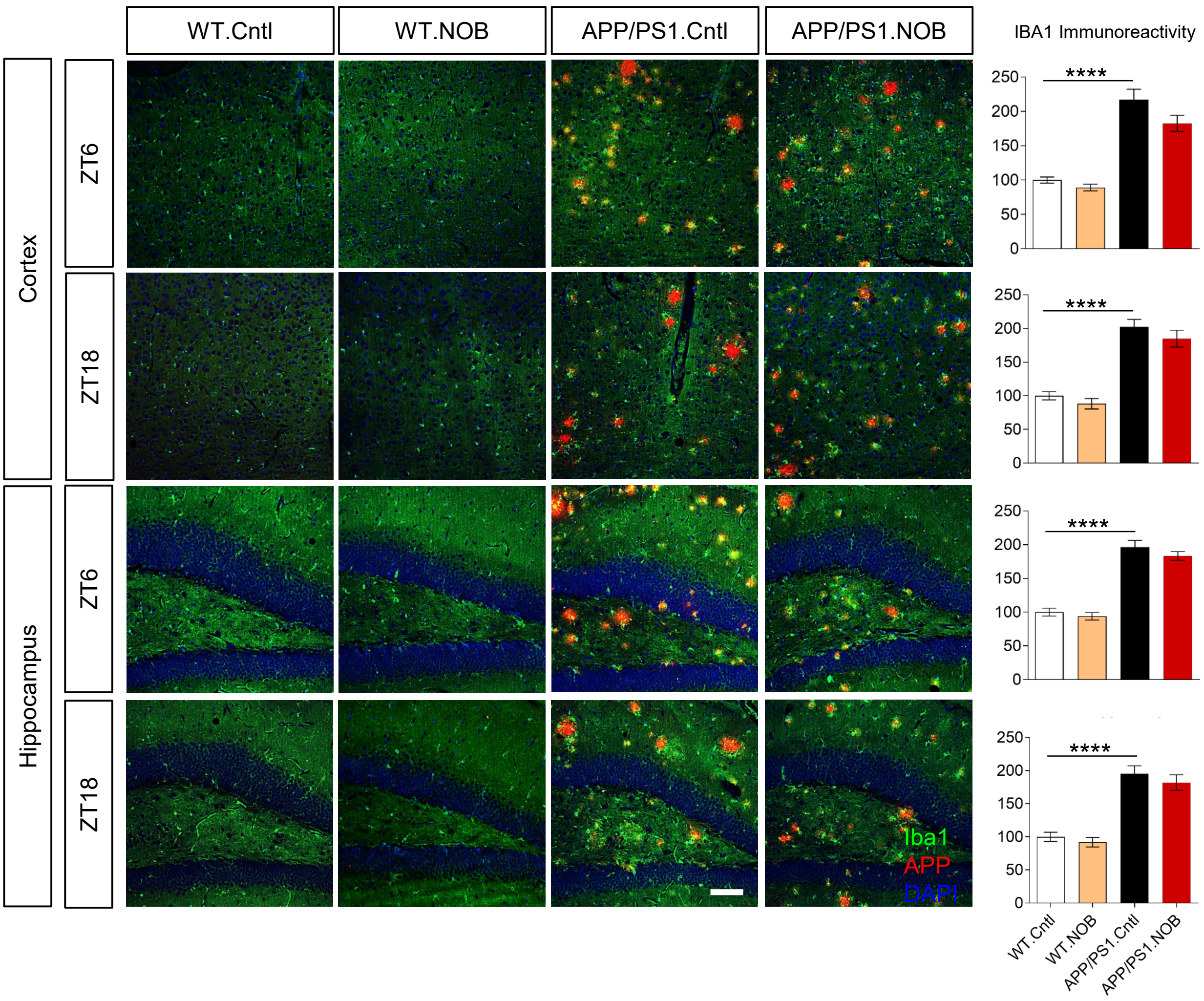

Figure S6. NOB did not alter immunoreactivity of the microgliosis marker IBA1 in APP/PS1 mice. Double immunofluorescence staining in the cortex (upper two rows) and the hippocampus (lower two rows) at two different time points (ZT6 and ZT18). Scale bar: 100 µm. Green: IBA1 (microglia); red: 4G8 (Aβ). Right panels: Quantification of IBA1 immunoreactivity. Data are presented as mean ± SEM. ****, $p<0.0001$: two-way ANOVA with Tukey's multiple comparisons.

Table S1. Primer sequences for RT-qPCR.

	Forward (5'-3')	Reverse (5'-3')
Clock	CCTTCAGCAGTCAGTCCATAAAC	AGACATCGCTGGCTGTGTTAA
Bmal1	CCACCTCAGAGCCATTGATACA	GAGCAGGTTTAGTTCCACTTTGTCT
Per1	TTCGTGGACTTGACACCTCTT	GGGAACGCTTTGCTTTAGAT
Per2	ATGCTCGCCATCCACAAGA	GCGGAATCGAATGGGAGAAT
Per3	AAAAGCACCACGGATACTGGC	GGGAGGCTGTAGCTTGTCA
Cry1	CTGGCGTGGAAGTCATCGT	CTGTCCGCCATTGAGTTCTATG
Cry2	TGTCCCTTCCTGTGTGGAAGA	GCTCCCAGCTTGGCTTGA
Npas2	CAACAGACGGCAGCATCATCT	TTCTGATCCATGACATCCGC
Rora	GCACCTGACCGAAGACGAAA	GAGCGATCCGCTGACATCA
Nr1d1	CATGGTGCTACTGTGTAAGGTGTGT	CACAGGCGTGCACTCCATAG
Dbp	CTGGCCCGAGTCTTTTTGC	CCAGGTCCACGTATTCCACG
App	AGCACCGAGAGAGAATGTCC	GCAAGTTCTTGGCTTGACG
Bace1	ACATTGCTGCCATCACTGAA	GCCTGGCAATCTCAGCATAG
Bace2	TGAGGACCTTGTCACCATCCCAAA	TGGCCAAAGCAGCATAAGCAAGTC
Apoe	ATTGCGAAGATGAAGGCTCT	CCACTCGAGCTGATCTGTCA
Scna	TGACAGCAGTCGCTCAGA	CATGTCTTCCAGGATTCCTTC
Scnb	GGAGGAGCTGTGTTCTCTGG	TCCTCTGGCTTCAGGTCTGT
Lrp1	ATTGAGGGCAAGATGACACA	CCAGTCTGTCCAGTACATCCAC
Adam10	ACAGACTTGGCTCTCGATAAACTT	GGTATGTACATTGGCAAGTGATGT
Atxn3	TGTCTTGTTACAGAAAGATCAG	GTTACAAGAACAGAGCTGACT
Atxn10	TCAGAGTGGCCGTTCTTGAT	ATCCTTTGTCAGCTGCTCCT
Tnfa	CTGTAGCCCACGTCGTAGC	TTGAGATCCATGCCGTTG
IL1b	TGTGGCAGCTACCTGTGTCT	TCATCTCGGAGCCTGTAGTG
IL6	TACCACTTCACAAGTCGGAGG	CTGCAAGTGCATCATCGTTGT
IL4	ACAGGAGAAGGGACGCCAT	GAAGCCCTACAGACGAGCTCA
IL17	GCTCCAGAAGGCCCTCAGA	AGCTTTCCCTCCGCATTGA
IL18	CAGGCCTGACATCTTCTGCAA	TCTGACATGGCAGCCATTGT
Ifngr	TCAAGTGGCATAGATGTGGAAGAA	TGGCTCTGCAGGATTTTCATG
Gapdh	CAAGGTCATCCATGACAACTTTG	GGCCATCCACAGTCTTCTGG

Table S2. Statistical significance and F distribution of interaction by three-way ANOVA for Figures 2 and S2. *, p<0.05; **, p<0.01; ***, p<0.001; ****, p<0.0001. The F distribution shows two parameters including degrees of freedom numerator (dfn) and degrees of freedom denominator (dfd).

Gene Name	P and F values	Treatment	Genotype	Time point	Treatment x Genotype	Timepoint X Treatment	Timepoint X Genotype	Timepoint X Treatment X Genotype
Clock	*P* value	ns	ns	ns	ns	*	**	*
	F(dfn,dfd)	F(1,26)=3.744	F(1,26)=1.349	F(1,26)=0.179	F(1,26)=3.744	F(1,26)=4.378	F(1,26)=10.54	F(1,26)=4.378
Bmal1	*P* value	***	ns	ns	*	****	ns	ns
	F(dfn,dfd)	F(1,26)=20.27	F(1,26)=0.749	F(1,26)=0.007	F(1,26)=6.694	F(1,26)=23.37	F(1,26)=1.816	F(1,26)=0.253
Rora	*P* value	****	*	*	****	ns	**	ns
	F(dfn,dfd)	F(1,26)=97.14	F(1,26)=7.589	F(1,26)=4.818	F(1,26)=29.93	F(1,26)=3.056	F(1,26)=9.485	F(1,26)=0.03
Nr1d1	*P* value	**	ns	ns	**	*	ns	ns
	F(dfn,dfd)	F(1,26)=8.073	F(1,26)=0.013	F(1,26)=1.688	F(1,26)=12.21	F(1,26)=6.129	F(1,26)=1.307	F(1,26)=0.698
Per1	*P* value	**	**	ns	ns	ns	ns	ns
	F(dfn,dfd)	F(1,26)=10.83	F(1,26)=11.57	F(1,26)=0.894	F(1,26)=0.423	F(1,26)=1.794	F(1,26)=0.497	F(1,26)=0.27
Per2	*P* value	**	ns	***	****	ns	ns	ns
	F(dfn,dfd)	F(1,26)=10.17	F(1,26)=0.677	F(1,26)=14.97	F(1,26)=21.81	F(1,26)=0.04	F(1,26)=0.229	F(1,26)=3.456
Per3	*P* value	ns	**	ns	ns	ns	ns	ns
	F(dfn,dfd)	F(1,26)=1.662	F(1,26)=13.11	F(1,26)=0.004	F(1,26)=0.565	F(1,26)=1.394	F(1,26)=3.315	F(1,26)=2.64
Npas2	*P* value	****	***	****	ns	***	*	ns
	F(dfn,dfd)	F(1,26)=28.93	F(1,26)=17.73	F(1,26)=55.44	F(1,26)=3.237	F(1,26)=18.61	F(1,26)=5.626	F(1,26)=2.252
Cry1	*P* value	***	ns	****	ns	ns	ns	ns
	F(dfn,dfd)	F(1,26)=13.74	F(1,26)=0.000	F(1,26)=94.67	F(1,26)=0.666	F(1,26)=0.002	F(1,26)=1.548	F(1,26)=0.004
Cry2	*P* value	ns	ns	**	ns	*	ns	ns
	F(dfn,dfd)	F(1,26)=0.588	F(1,26)=0.014	F(1,26)=11.97	F(1,26)=3.45	F(1,26)=5.667	F(1,26)=2.053	F(1,26)=2.407
Dbp	*P* value	**	ns	ns	**	***	*	ns
	F(dfn,dfd)	F(1,26)=12.40	F(1,26)=0.085	F(1,26)=0.451	F(1,26)=11.37	F(1,26)=20.42	F(1,26)=5.554	F(1,26)=0.779
App	*P* value	****	****	ns	ns	ns	ns	*
	F(dfn,dfd)	F(1,26)=25.98	F(1,26)=50.73	F(1,26)=3.189	F(1,26)=3.361	F(1,26)=0.177	F(1,26)=0.813	F(1,26)=6.722
Bace1	*P* value	***	ns	ns	**	ns	ns	ns
	F(dfn,dfd)	F(1,26)=18.44	F(1,26)=3.308	F(1,26)=0.389	F(1,26)=12.24	F(1,26)=0.407	F(1,26)=1.246	F(1,26)=0.000
Bace2	*P* value	ns	ns	**	**	*	ns	**
	F(dfn,dfd)	F(1,26)=1.230	F(1,26)=0.268	F(1,26)=8.188	F(1,26)=9.904	F(1,26)=6.050	F(1,26)=1.337	F(1,26)=8.625
Apoe	*P* value	**	ns	*	**	ns	ns	ns
	F(dfn,dfd)	F(1,26)=12.51	F(1,26)=0.521	F(1,26)=6.041	F(1,26)=12.81	F(1,26)=0.203	F(1,26)=0.030	F(1,26)=0.630
Scna	*P* value	**	ns	*	****	ns	ns	***
	F(dfn,dfd)	F(1,26)=11.05	F(1,26)=0.101	F(1,26)=5.199	F(1,26)=29.13	F(1,26)=2.504	F(1,26)=1.285	F(1,26)=19.89
Scnb	*P* value	ns	****	**	**	ns	ns	ns
	F(dfn,dfd)	F(1,26)=1.606	F(1,26)=21.16	F(1,26)=9.328	F(1,26)=9.568	F(1,26)=1.385	F(1,26)=2.477	F(1,26)=1.675
Lrp1	*P* value	****	****	**	*	ns	**	*
	F(dfn,dfd)	F(1,26)=27.32	F(1,26)=37.40	F(1,26)=12.95	F(1,26)=6.008	F(1,26)=0.231	F(1,26)=11.41	F(1,26)=4.758
Adam10	*P* value	****	ns	****	*	**	ns	ns
	F(dfn,dfd)	F(1,26)=23.57	F(1,26)=0.362	F(1,26)=45.1	F(1,26)=6.991	F(1,26)=9.9	F(1,26)=0.184	F(1,26)=0.755
Axtn3	*P* value	ns	*	ns	ns	ns	ns	ns
	F(dfn,dfd)	F(1,26)=1.377	F(1,26)=7.2	F(1,26)=2.993	F(1,26)=3.88	F(1,26)=0.169	F(1,26)=0.007	F(1,26)=3.029
Axtn10	*P* value	ns	**	ns	ns	**	ns	****
	F(dfn,dfd)	F(1,26)=0.028	F(1,26)=9.555	F(1,26)=0.617	F(1,26)=3.305	F(1,26)=11.83	F(1,26)=0.01	F(1,26)=22.59

Table S3. Statistical significance and F distribution of interaction by three-way ANOVA for Figure 4. *, p<0.05; **, p<0.01; ***, p<0.001; ****, p<0.0001. The F distribution shows two parameters including degrees of freedom numerator (dfn) and degrees of freedom denominator (dfd).

Gene and Protein Name	P and F values	Treatment	Genotype	Time point	Treatment x Genotype	Timepoint X Treatment	Timepoint X Genotype	Timepoint X Treatment X Genotype
Tnfa	*P* value	****	**	ns	*	ns	**	ns
	F(dfn,dfd)	F(1,26)=32.77	F(1,26)=8.358	F(1,26)=1.562	F(1,26)=7.359	F(1,26)=0.87	F(1,26)=10.13	F(1,26)=4.137
IL1b	*P* value	****	**	ns	ns	ns	*	ns
	F(dfn,dfd)	F(1,26)=24.37	F(1,26)=11.90	F(1,26)=0.017	F(1,26)=2.276	F(1,26)=0.033	F(1,26)=6.743	F(1,26)=1.503
IL6	*P* value	****	***	ns	**	ns	*	ns
	F(dfn,dfd)	F(1,26)=40.42	F(1,26)=14.94	F(1,26)=1.184	F(1,26)=13.37	F(1,26)=0.286	F(1,26)=5.182	F(1,26)=2.757
IL4	*P* value	**	***	ns	***	ns	ns	ns
	F(dfn,dfd)	F(1,26)=11.53	F(1,26)=19.98	F(1,26)=0.464	F(1,26)=20.83	F(1,26)=0.106	F(1,26)=0.011	F(1,26)=0.278
IL17	*P* value	**	*	ns	**	ns	ns	*
	F(dfn,dfd)	F(1,26)=12.69	F(1,26)=5.324	F(1,26)=0.007	F(1,26)=8.611	F(1,26)=0.019	F(1,26)=0.992	F(1,26)=5.175
IL18	*P* value	****	***	ns	**	ns	ns	ns
	F(dfn,dfd)	F(1,26)=30.46	F(1,26)=16.81	F(1,26)=0.589	F(1,26)=12.16	F(1,26)=0.066	F(1,26)=1.329	F(1,26)=0.309
Ifngr	*P* value	**	****	**	**	ns	ns	ns
	F(dfn,dfd)	F(1,26)=10.23	F(1,26)=35.08	F(1,26)=8.707	F(1,26)=10.53	F(1,26)=0.6	F(1,26)=1.780	F(1,26)=0.262
NLRP3	*P* value	*	****	ns	ns	ns	****	ns
	F(dfn,dfd)	F(1,26)=8.521	F(1,26)=260.9	F(1,26)=1.252	F(1,26)=1.654	F(1,26)=0.008	F(1,26)=69.01	F(1,26)=4.448
